# Differential Contributions of Olfactory Receptor Neurons in a Drosophila Olfactory Circuit

**DOI:** 10.1523/ENEURO.0045-16.2016

**Published:** 2016-07-28

**Authors:** Gunnar Newquist, Alexandra Novenschi, Donovan Kohler, Dennis Mathew

**Affiliations:** Department of Biology, University of Nevada, Reno, Nevada 89557

**Keywords:** *Drosophila*, *larva*, *olfaction*, *receptor neuron*, *odor receptor*, *behavior*

## Abstract

The ability of an animal to detect, discriminate, and respond to odors depends on the functions of its olfactory receptor neurons (ORNs). The extent to which each ORN, upon activation, contributes to chemotaxis is not well understood. We hypothesized that strong activation of each ORN elicits a different behavioral response in the *Drosophila melanogaster* larva by differentially affecting the composition of its navigational behavior. To test this hypothesis, we exposed *Drosophila* larvae to specific odorants to analyze the effect of individual ORN activity on chemotaxis. We used two different behavioral paradigms to analyze the chemotaxis response of larvae to odorants. When tested with five different odorants that elicit strong physiological responses from single ORNs, larval behavioral responses toward each odorant differed in the strength of attraction as well as in the composition of discrete navigational elements, such as runs and turns. Further, behavioral responses to odorants did not correlate with either the strength of odor gradients tested or the sensitivity of each ORN to its cognate odorant. Finally, we provide evidence that wild-type larvae with all ORNs intact exhibit higher behavioral variance than mutant larvae that have only a single pair of functional ORNs. We conclude that individual ORNs contribute differently to the olfactory circuit that instructs chemotactic responses. Our results, along with recent studies from other groups, suggest that ORNs are functionally nonequivalent units. These results have implications for understanding peripheral odor coding.

## Significance Statement

Olfactory behavior in the *Drosophila* larva is based on the activities of only 21 olfactory receptor neurons (ORNs). An intriguing question in the biology of sensory systems concerns the functional diversity among its ORNs. Through systematic olfactory behavior analyses, we report that the activation of each larval ORN differently influences discrete navigational elements such as runs and turns. One interpretation is that individual ORNs contribute differently to the olfactory circuit that leads to chemotactic response. This analysis of functional diversity among ORNs has implications for developing more reliable models of odor coding.

## Introduction

Sophisticated olfactory function in the *Drosophila* larva is based on the activities of only 21 first-order sensory neurons known as olfactory receptor neurons (ORNs). ORNs innervate the dorsal organ of the head and send axons to glomeruli in the larval antennal lobe (Fishilevich et al., 2005; Ramaekers et al., 2005; Kreher et al., 2008). Within each glomerulus, input from a single ORN is received by a second-order projection neuron, which in turn relays information to higher olfactory centers in the brain (Masuda-Nakagawa et al., 2005, 2009,2010; Ramaekers et al., 2005). Subsequent processing of information in higher olfactory centers instructs olfactory behavior responses of the larva. Thus, the 21 ORNs constitute discrete information-processing channels in the larval brain. While a considerable amount of information has been generated regarding sensory neuron responses to odorants, much less is known about the functional diversity among sensory neuron channels and its role in driving behavioral output (Fishilevich et al., 2005; Kreher et al., 2008; Louis et al., 2008; Montague et al., 2011; Mathew et al., 2013). Two recent studies (Mathew et al., 2013; Hernandez-Nunez et al., 2015) suggested that the activation of different chemosensory neurons in the *Drosophila* larva could produce behavioral responses of different strengths or dynamics. However, these studies either used a simple behavioral assay to measure response strength or considered only a single aspect of chemotactic navigation behavior to measure response dynamics. The main objective of this research is to unequivocally establish that there is diversity in the functional contributions of individual larval sensory neurons to complex olfactory behavior.

The activities of the ORNs are based on the responses of odor receptors (Ors). Larval ORNs together express 25 members of the Or family of odor receptors and the Or coreceptor (Orco) (Couto et al., 2005; Fishilevich and Vosshall, 2005; Kreher et al., 2005, 2008). In each ORN, Or and Orco proteins together form a ligand-gated ion channel (Sato et al., 2008; Smart et al., 2008; Wicher et al., 2008). Most ORNs, with the exception of a few cases, express a single Or (Fishilevich et al., 2005; Kreher et al., 2005). Recent studies have characterized the odor response profile of every larval Or in terms of its breadth of tuning, receptor sensitivity, and temporal dynamics (Kreher et al., 2005, 2008; Mathew et al., 2013).

A panel of odorants that elicit strong (>150 spikes/s) and specific physiological responses from 19 of 21 larval ORNs was recently identified (Mathew et al., 2013). This panel of strong ORN activators, when tested in a simple two-choice behavioral paradigm, drove behavioral responses that varied across a continuum. One hypothesis is that differences in behavior could arise due to differences in strengths of the odor gradients formed and/or due to differences in Or sensitivities with their cognate odorant. An alternate hypothesis to account for the differences in behavior elicited by strong ORN activators is that individual ORNs contribute differently to information processing in the olfactory circuit. Testing these hypotheses requires precise stimulus delivery methods and extensive behavior analyses that can create a more complete picture of behavior driven by activation of each ORN.

**Fig. 1.**
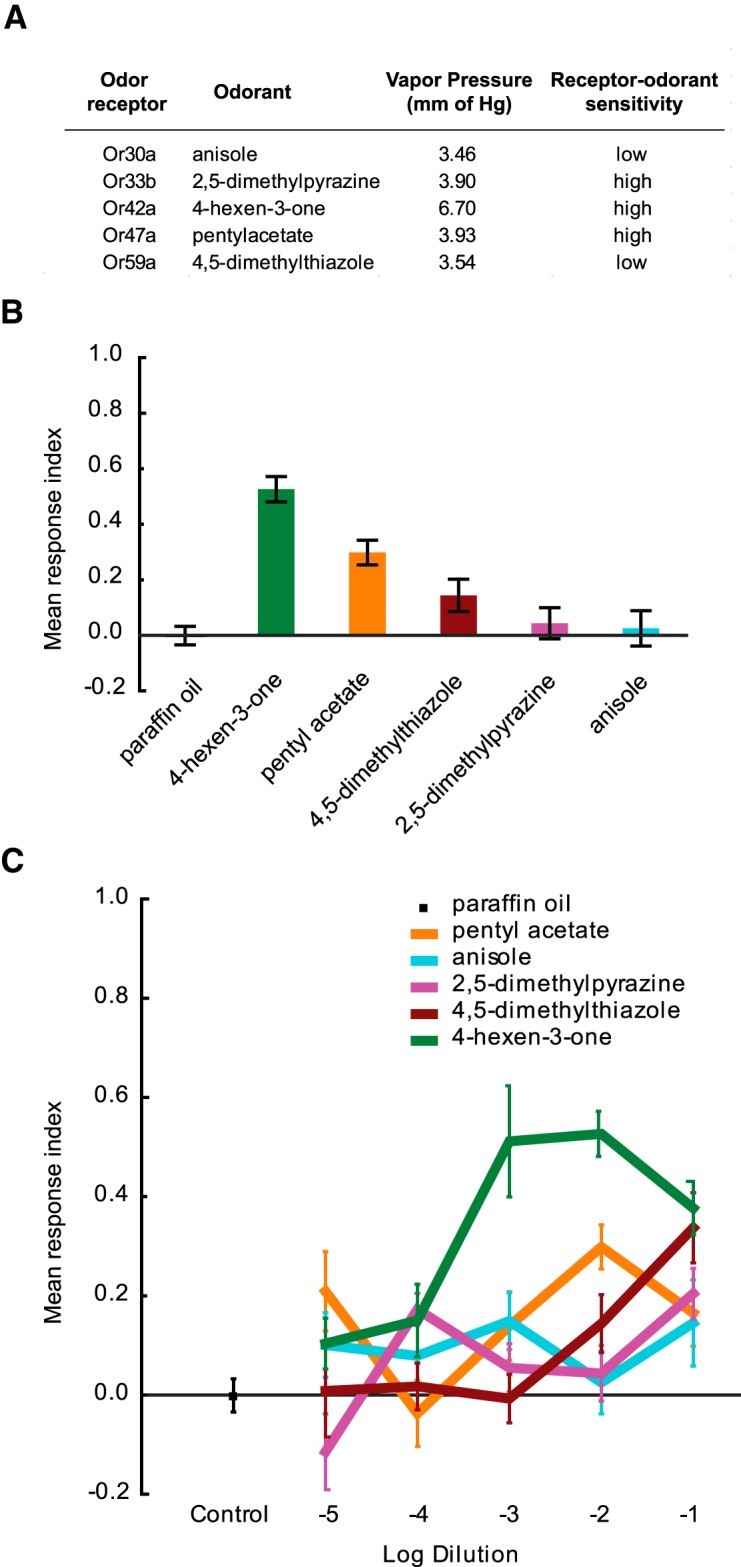
*Drosophila melanogaster* larvae respond differently to odorants activating individual ORNs in a two-choice small-format paradigm. ***A***, Five odorants selected for this study are shown. Listed next to each odorant is the odor receptor that it activates, its vapor pressure measured in millimeters of mercury at 25°C, and the sensitivity of each odor receptor to its cognate odorant determined in an electrophysiology assay (Mathew et al., 2013). ***B***, Mean RIs of wild-type *Drosophila* larvae tested in the presence of odorants in a two-choice behavior paradigm are shown. Odorants were tested at a 10^−2^ dilution. Each bar represents the RI ± SEM (*n* = 8). Responses differ; for example, the response to anisole differs from the responses to 4-hexen-3-one and pentyl acetate (Tukey’s HSD within a one-way ANOVA, *p* < 0.001). ***C***, Dose–response analysis for each odorant in the two-choice behavior paradigm. Odorants were tested at five different dilutions (10^−1^, 10^−2^, 10^−3^, 10^−4^, and 10^−5^). Each data point represents the RI ± SEM (*n* = 8).

In this study, we compare the behavioral responses of *Drosophila* larva to five odorants (Fig. 1*A*). These five odorants were chosen because (1) each elicits a strong, specific response from a different odor receptor and shows little cross-activation of other receptors in a physiological test, (2) all five odorants have similar volatilities and form odor gradients of similar strengths, and (3) three of the five odor receptors activated by this panel of odorants exhibit similar sensitivities to their cognate odorant (Mathew et al., 2013). To quantify larval migration toward or away from odorants, we use a classic behavior assay (Rodrigues and Siddiqi, 1978; Monte et al., 1989). To conduct more extensive analyses of larval behavior and define olfactory computations, we perform quantitative behavioral analyses with the help of a larval tracking paradigm (Gershow et al., 2012; Mathew et al., 2013).

Here, we address whether individual ORNs contribute differently to the olfactory circuit by asking the following three specific questions. Does the activity of individual ORNs elicit different behavioral responses? Does the activity of individual ORNs differentially affect the composition of navigational behavior? Are the contributions of individual ORNs to olfactory behavior different when its neighboring neurons are silent? To understand the transformation of olfactory information into larval navigation, it is necessary to understand the contributions of individual sensory neurons to the olfactory circuit.

## Materials and Methods

### *Drosophila* stocks

A *Canton-S* (CS) line was used as the wild-type line in behavioral experiments. The *Orco^1^* mutant (*Drosophila* Stock Center, Bloomington, IN), which was backcrossed to a *wCS* line for 10 generations, was used to generate the three empty larva genotypes (Fishilevich et al., 2005). Females from a *UAS-Orco; Orco^1^* were crossed to males from an *OrX-Gal4; Orco^1^* line (where *X* = Or30a/42a/47a). F1 progeny from this cross were used for the empty larva experiments.

### Odorants and other reagents

Odorants used in these studies were obtained at the highest purity available (≥98% purity; Sigma-Aldrich). They were diluted in paraffin oil (Sigma-Aldrich) for our studies. High-purity Agarose [Apex Bioresearch (purchased from Genesee Scientific Inc.)] gel was used to prepare the crawling surface for larvae during chemotaxis behavior experiments. The 6 mm filter discs (GE Whatman) used in the behavior assays were purchased from VWR Inc.

### Behavioral assays

#### Preparation of larvae for behavior assays

Third-instar larvae (∼96 h after egg laying) are extracted from food using a high-density (15%) sucrose (Sigma-Aldrich) solution. Larvae that float to the surface of the sucrose solution are separated into a 1000 ml glass beaker and washed four times with distilled water. Washed larvae are allowed to rest for 10 min before subjecting them to behavior assays. The temperature of the behavior room is maintained between 22°C and 23°C. The humidity of the room is maintained at between 45% and 50% relative humidity.

A two-choice assay was conducted as described previously (Monte et al., 1989; Kreher et al., 2008). Briefly, odor was added to a filter disc on one side of a 9 cm Petri dish, and the diluent (paraffin oil) was added to a filter disc on the opposite side. Approximately 50 third-instar larvae were placed in the center of the dish and allowed 5 min to migrate. After 5 min, the number of larvae on each half of the dish was counted to generate the response index (RI).

A tracking assay was conducted as described previously (Mathew et al., 2013). Briefly, odor was added to five filter discs placed equidistant from each other against one wall of a 22 × 22 cm^2^ Petri dish layered with 1.5% agarose. Control diluent was added to five filter discs placed against the opposite wall. Approximately 20 third-instar larvae were placed in the center of the dish along a line parallel to the discs. Larvae were imaged within the experimental arena under dark-field illumination with infrared LEDs (850 nm, outside the range of larval phototaxis; Environmental Lights). Images were recorded at 2.3 frames/s using a Monochrome USB 3.0 camera (Basler Ace series, JH Technologies) fitted with an IR long-pass 830 nm filter and an 8 mm F1.4 C-mount lens (JH Technologies). Each pixel in the captured image corresponded to a 0.119 mm^2^ of the experimental arena.

### Data processing and statistical analysis

#### Navigational parameters

For analyzing larval navigation in the tracking assay, positions of larvae for the entire duration of the assay were extracted from video recordings, and larval “trajectories” were reconstructed by using custom routines written in MATLAB (MathWorks; RRID: SCR_001622). Eighty to 120 trajectories were analyzed for each experiment. Wild-type larvae displayed an average trajectory length of 176.33 ± 2.8 mm for the duration of the tracking assay. No significant differences were observed among average trajectory lengths for any of the test conditions (ANOVA, *p* > 0.05). The navigational index *<v_x_>/<s>* was defined as the mean velocity of the larva in the *x* direction (*<v_x_>*) divided by the mean crawling speed (*<s>*), as described previously by Gershow et al. (2012). Based on some navigational statistics, such as speed, path curvature, and heading angle, we segmented trajectories into alternating sequences of runs and turns. Runs were defined as continuous periods of forward movement. Turns separated successive runs. Turns were flagged when the change of trajectory orientation angle was >45°. Further statistics were applied to individual runs to calculate run direction (average orientation of runs in a scale of 0 to ±180, with “0” → toward the odor and ±180 → away from the odor), run length, and run speed. Run length and run speed were further calculated for runs (toward) odor (all runs that oriented between +45° and −45°) and for runs (away) from odor (all runs that oriented between +135° and −135°). The run ratio was calculated as the mean run length of runs toward odor divided by the mean run length of runs away from odor. Path curvature was defined as the total length of a trajectory divided by its total displacement.

#### Principal component analysis

Principal component analysis (PCA) of behavior spaces (see Figs. 3*A*, 5*A* ) was performed with built-in MATLAB functions. A behavior space was constructed using nine navigational descriptors [RI, number of runs per trajectory, run length (toward), run length (away), run speed (toward), run speed (away), run ratio, run direction, and length/displacement]. For all genotypes, only data from the 10^−2^ dilution were considered. Descriptors were normalized by dividing the value of each descriptor by its variance: normalized descriptor = descriptor/variance. Euclidean distances were calculated using MATLAB functions.

#### Statistics

**Fig. 2.**
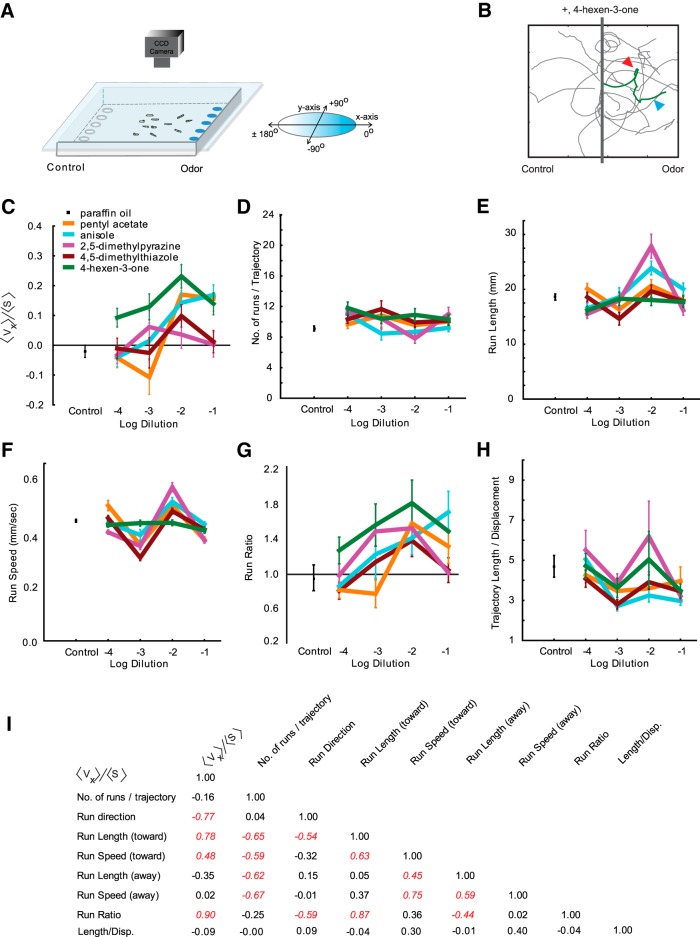
Navigational analysis of wild-type *D. melanogaster* larvae. ***A***, Paradigm containing a 22 × 22 cm^2^ agarose Petri plate. Odorant is placed on discs at the right; paraffin oil diluent alone is placed on discs to the left. The chamber is sealed by placing a clear glass plate over the arena. Third-instar larvae are placed in the center. The movement of larvae is recorded with a CCD camera. ***B***, Sample trajectories of wild-type larvae in response to 4-hexen-3-one (10^−2^ dilution). The gray bar along the *y*-axis indicates the starting position of larvae. A “stop” (red arrowhead) is defined by a 45° or greater change in trajectory angle. A “run” (blue arrowhead) is defined as the length of trajectory between two stops. Runs are quantified in terms of length, speed, and direction. ***C–H***, Dose–response analysis of six navigational parameters for each odorant in the navigational assay. Odorants were tested at four different dilutions (10^−1^, 10^−2^, 10^−3^, 10^−4^), which are depicted on the *x*-axis of each graph. The *y*-axes in each graph are as follows: navigational indices (*<v_x_>/<s>*) of larvae to indicated dilutions of five odorants and paraffin oil (***C***); the mean number of runs per trajectory (***D***); the mean length of runs in millimeters (***E***); the mean speed of runs in millimeters per second (***F***); the ratio of mean run lengths in the direction of odorant (all runs that oriented between +45° and −45°) to mean run lengths away from odorant (all runs that oriented between +135° to −135°; ***G***); and the mean length/displacement defined as total length of each trajectory is divided by the total displacement of each trajectory (***H***). Each data point represents the mean ± SEM (*n* = 8 assays, ∼100–120 trajectories analyzed for each condition). ***I***, Correlation matrix displaying *r* ^2^ values among various behavioral parameters tested. Values italicized and in red are statistically significant (*p* < 0.05).

Statistical analyses were performed using Statistica (StatSoft; RRID: SCR_014213; Table 1). In all figures except Figure 2*G*(and see Fig. 4*G*) the plotted error bars are SEM values. For error bars in Figure 2*G*(and see Fig. 4*G*), uncertainties (*dr*) in “run ratio” values were calculated using the following formula: $dr=R\times \sqrt{{\left(dt/T\right)}^{2}+{\left(da/A\right)}^{2}},$ where *T* is the average run length (toward), *A* is average run length (away), *R* is the run ratio (*T/A*), and *dt* and *da* are the respective uncertainties of *T* and *A*.

For correlation analyses in Figure 2*I*(and see Fig. 4*I*), *r* ^2^ values were calculated using Pearson’s correlation matrix. Statistical significance was set at the 0.05 level.

**Table 1:** Summary of statistics from figures

Figure	Panel	Data structure	Test type	Odorant	*p* value
1	*A*	Normal	Tukey HSD within a one-way ANOVA	4-Hexen-3-one	*p* < 0.001
			Tukey HSD within a one-way ANOVA	Pentyl acetate	*p* < 0.001
			Tukey HSD within a one-way ANOVA	4,5-Dimethylthiazole	*p* = 0.2199
			Tukey HSD within a one-way ANOVA	2,4-Dimethylpyrazine	*p* = 0.9811
			Tukey HSD within a one-way ANOVA	Anisole	*p* = 0.9985
2	*I*	Report *r*^2^ values	Pearson's correlation matrix	All combinations	*p* < 0.05 highlighted in red
3	*A*/*B*	Euclidean distance	Principal component analysis	All combinations	NA
4	*I*	Report *r*^2^ values	Pearson's correlation matrix	All combinations	*p* < 0.05 highlighted in red
5	*A*/*B*	Euclidean distance	Principal component analysis	All combinations	NA
6	*A*/*B*	Normal	Tukey HSD within a one-way ANOVA	Vx/S, all odorants, 10^−1^, 10^−2^, 10^−3,^	Dark red: *p* < 0.001
6	*A*/*B*	Ratio	Χ^2^ normalized to control	Run Ratio, all odorants, 10^−1^, 10^−2^, 10^−3,^	Red: *p* < 0.01
6	*A*/*B*	Normal	Tukey HSD within a one-way ANOVA	Run length, all odorants, 10^−1^, 10^−2^, 10^−3,^	Light red: *p* < 0.05
6	*A*/*B*	Normal	Tukey HSD within a one-way ANOVA	Run speed, all odorants, 10^−1^, 10^−2^, 10^−3,^	Dark blue: *p* < 0.001
6	*A*/*B*	Normal	Tukey HSD within a one-way ANOVA	Length/Disp, all odorants, 10^−1^, 10^−2^, 10^−3,^	Blue: *p* < 0.01
6	*A*/*B*	Normal	Tukey HSD within a one-way ANOVA	Runs/track, all odorants, 10^−1^, 10^−2^, 10^−3,^	Light blue: *p* < 0.05
6	*C*	Normal	Student's *t* test with Bonferroni correction	Vx/S, 10^−1^ to 10^−3^	*p* < 0.01
6	*C*	Normal	Student's *t* test with Bonferroni correction	Run length, 10^−1^ to 10^−3^	*p* < 0.001
6	*C*	Normal	Student's *t* test with Bonferroni correction	Run speed, 10^−1^ to 10^−3^	*p* < 0.001
6	*C*	Normal	Student's *t* test with Bonferroni correction	Length/Disp, 10^−1^ to 10^−3^	*p* = 0.4549
6	*C*	Normal	Student's *t* test with Bonferroni correction	Runs/track, 10^−1^ to 10^−3^	*p* = 0.0411

Statistical analyses were performed using Statistica (StatSoft). Kolmogorov–Smirnov test was used to test for normality within each group.

For all behavioral parameters (except for run ratio) seen in Figure 6, *A* and *B*, a one-way ANOVA followed by a Tukey’s *post hoc* HSD test and a homogeneity of variance test were performed to compare values elicited by the odorant to values elicited by paraffin oil. Statistical significance for the ANOVA was set at *p* = 0.05. For the run ratio, values were normalized and subjected to a χ^2^ test followed by a Bonferroni correction. Since 15 tests were performed for wild type and only 12 were performed for empty larva genotypes, statistical significance for the χ^2^ test was set at *p* = 0.0033 for wild-type larvae and *p* = 0.0042 for empty larvae.

For comparison of SDs in Figure 6*C*, a Student’s *t* test was used followed by Bonferroni correction. Data for five behavioral parameters at 10^−1^, 10^−2^, and 10^−3^ dilutions were compared. Statistical significance for the *t* test was set at *p* = 0.01. Data were scaled to fit on a graph.

## Results

### Individual ORNs instruct different attractive responses

In a recent study, a panel of 18 odorants, each of which elicited a strong, specific physiological response from a single Or, was identified. A strong physiological response, however, did not always elicit a strong behavioral response in the larva (Mathew et al., 2013). Based on these results, we postulated that individual ORNs contribute differently to the olfactory circuit to produce distinct behavioral outputs. However, differences in behavior could also arise because of differences in the strengths of the odor gradient formed and/or due to differences in Or sensitivities to their cognate odorant. We wished to ask whether larval ORNs drive different behavioral responses under comparable odor gradient strengths and odor receptor sensitivities. To begin to address this question, we selected a subset of five odorants from the panel of 18 odorants published in the study by Mathew et al. (2013; Fig. 1*A*). Each of the five odorants elicits a strong, specific physiological response from a single Or (Or30a :: anisole, Or33b :: 2,5-dimethylpyrazine, Or42a :: 4-hexen-3-one, Or47a :: pentyl acetate, Or59a :: 4,5-dimethylthiazole). We selected this subset of five odorants since they have similar volatilities (average vapor pressure of five test odorants is 4.30 ± 1.35 mm of Hg at 25°C), and, thus, in a test arena, they form odor gradients of similar strengths. Further, in a dose–response analysis, three of the five Ors (33b, 42a, and 47a) showed high (but similar) sensitivities to their cognate odorant, while the other two Ors (30a and 59a) exhibited lower (but similar) sensitivities to their cognate odorants (Mathew et al., 2013). Thus, this subset of odorants presented a unique opportunity to test ORN activity under conditions of normalized odor gradient slopes and Or sensitivities.

First, we tested the response of wild-type larvae to this subset of five odorants in a simple two-choice assay behavioral paradigm (Rodrigues and Siddiqi, 1978; Monte et al., 1989). Briefly, ∼50 third-instar larvae are placed in the middle of an agarose Petri plate of 9 cm diameter. Two filter discs are placed diametrically opposed to one another, with one disc containing a drop of odorant (diluted to the test concentration) and the other serving as a control. Larvae are allowed to migrate onto the plate, and, after a 5 min test period, the number on each half is counted, and an RI is calculated as RI = (S − C)/(S + C), where *S* is the number on the half of the plate containing odorant and *C* is the number on the half containing the control disc. We note that the doses used in such a behavioral assay are difficult to compare with those used in the physiological assay in the study by Mathew et al. (2013) as a result of differences in airflow, duration, and geometry.

The data from this study are presented in Figure 1. When tested at a 10^−2^ dilution, each odorant elicited behavior responses of different strengths (Fig. 1*B*). 4-Hexen-3-one elicited the strongest attractive response, 0.53 ± 0.046 (SEM; *n* = 8). Pentyl acetate elicited a weaker attractive response (0.29 ± 0.045, SEM; *n* = 8), while 4,5-dimethylthiazole, 2,5-dimethylpyrazine, and anisole elicited responses that were not significantly different from zero. Paraffin oil, used as a control for the diluent, elicited a response that was not significantly different from zero (−0.002 ± 0.031; SEM; *n* = 21). When tested across five different dilutions (10^−1^, 10^−2^, 10^−3^, 10^−4^, 10^−5^), the five odorants generated dose–response curves that varied widely from each other (Fig. 1*C*). For instance, 4-hexen-3-one elicited strong attractive responses at higher concentrations (10^−1^, 10^−2^, and 10^−3^) and weaker responses at lower concentrations (10^−4^, 10^−5^). In contrast, pentyl acetate elicited an attractive response at 10^−2^ concentration of the compound but lost its attractiveness when its concentration was dropped by a single order of magnitude. Together, these data strongly suggest that behavioral differences exist despite similar odor gradient strengths and Or sensitivities. We conclude from these experiments that larval ORNs contribute differentially to the olfactory circuit to instruct the level of attraction to an odorant in the two-choice assay.

### Individual ORNs contribute differently to larval navigation

If activating individual ORNs elicits different levels of attraction in a two-choice assay, we postulated that the activity of individual ORNs generate different navigational outcomes toward or away from an odor. Recent studies have suggested that *Drosophila* larval navigation is composed of discrete behavioral elements, such as head sweeps, runs, and turns (Luo et al., 2010; Gomez-Marin et al., 2011; Gershow et al., 2012). We wished to ask whether different ORN activity led to different compositions of navigational behavior.

To address this question, we used a second behavioral paradigm, a larval tracking assay, which permits analysis of larval navigation. Briefly, ∼20 third-instar larvae are allowed to migrate toward an odor source on a square 22 × 22 cm^2^ agarose plate (Fig. 2*A*). Five filter discs containing odorant are placed at even intervals along one wall of the plate, and five filter discs containing a control diluent are placed at even intervals along the opposite wall. A CCD camera records the movement of the larvae for 5 min, and their positions are analyzed as a function of time (Mathew et al., 2013). Every larval trajectory in an experiment is divided into runs and turns (defined in Materials and Methods), and analyzed in terms of its speed, directionality, and displacement (Fig. 2*B*).

First, to quantify attraction toward an odorant, we measured the navigational index *<v_x_>/<s>* (Gershow et al., 2012), in which the mean velocity of larvae in the *x* direction, *<v_x_>*, is divided by the mean crawling speed *<s>*. Thus, the index is 1 if all larvae migrate uniformly toward the odor source, and 0 if their movement is random. The movement of wild-type larvae in the presence of paraffin oil diluent alone is not significantly different from zero and is random (−0.01 ± 0.02, SEM; *n* = 297 trajectories). When tested across four different dilutions (10^−1^, 10^−2^, 10^−3^, 10^−4^), the five odorants generated dose–response curves that varied widely from each other (Fig. 2*C*), which is consistent with the two-choice assay. 4-Hexen-3-one, pentyl acetate, and anisole elicited attractive indices at higher concentrations (10^−1^ and 10^−2^), and weaker or no attraction at lower concentrations (10^−3^, 10^−4^). On the other hand, 2,5-dimethylpyrazine and 4,5-dimethylthiazole elicited weak or no attractive response at all four concentrations tested in the assay.

The ability to measure larval behavior in terms of its discrete navigational elements allowed us to ask whether the navigational index is a sufficient measure of overall behavior response. To address this question, we examined five different navigational parameters (number of runs per trajectory, run length, run speed, run length toward odor/run length away from odor, and length of trajectory/total displacement) for all the trajectories generated in this assay (Gershow et al., 2012; Mathew et al., 2013; Gomez-Marin and Louis, 2014). For each of the five odorants, we plotted average values for each navigational parameter as a function of concentration (Fig. 2*D–H*). Dose–response curves for the five navigational measures reveal differences in larval behavior elicited by the individual odorants that are not apparent when considering only an attractive index (Figs. 1*B*,*C*, 2*C* ). Notably, 2,5-dimethylpyrazine, an odorant that elicits a weak attractive response, elicits significantly higher values for “run length” (Fig. 2*E*) and “run speed” (Fig. 2*F*) when compared to 4-hexen-3-one, an odorant that elicits a strong attractive response. We observed that four of the five dose–response traces for run speed follow a unique pattern (Fig. 2*F*). This is likely due to a number of reasons: (1) strong correlation between run speed and run length (toward odor) at the most attractive concentration of odorants (10^−2^; Fig. 2*E*; data not shown); and (2) higher mean speeds measured at low/ineffective odor concentrations (10^−4^) due to equally long run lengths both toward and away from odorants (data not shown). To further ask whether any of the navigational parameters elicited correlated with the attractive index, we prepared a correlation matrix consisting of correlation values (*r* ^2^) between individual behavioral parameters (Fig. 2*I*, Table 1). Values highlighted in red are statistically significant (*p* < 0.05). We found that for wild-type larvae the attractive index correlates with only four of eight behavioral parameters considered here. Overall, few parameters correlated with each other (16 of the possible 36 combinations). We note that run ratio (run length toward odor/run length away from odor) correlated strongly with the attractive index (*r* ^2^ = 0.90; Fig. 2*I*). On the other hand, speed ratio (run speed toward odor/run speed away from odor) correlated only moderately with the attractive index (*r* ^2^ = 0.71; data not shown). Overall, these observations suggested the following: (1) that attractive indices are an insufficient measure of overall larval behavioral response; (2) that larvae strongly modulate the lengths of their runs and to a lesser extent their speed in order to successfully navigate toward an odor; and (3) individual ORN activity can differentially affect the composition of larval navigation made up of discrete behavioral elements.

**Fig. 3.**
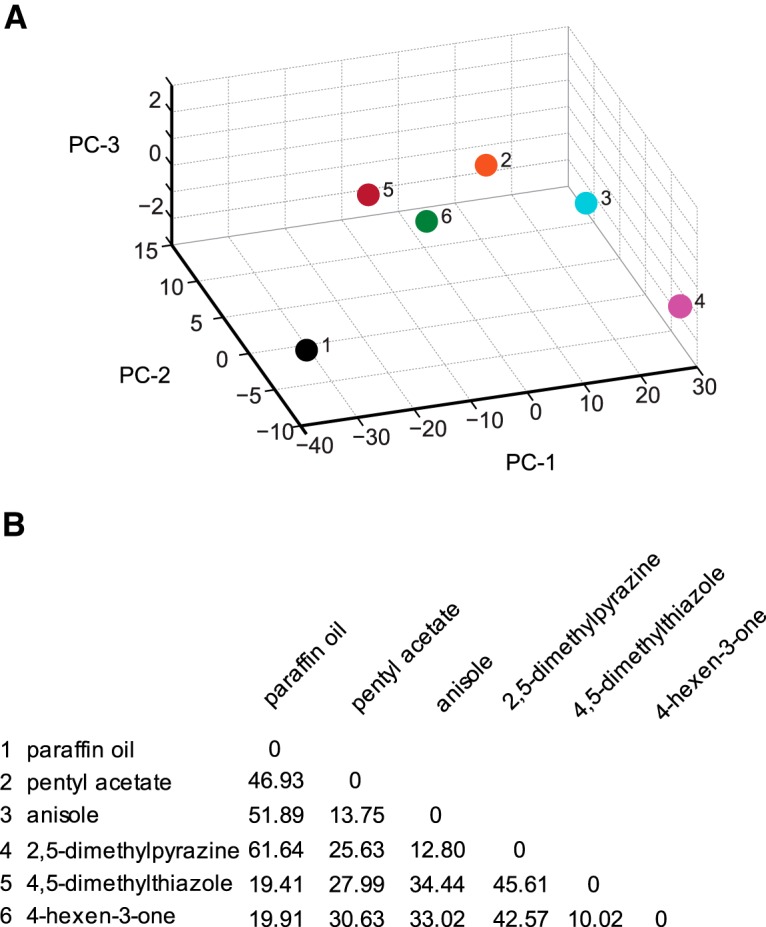
Principal component analysis of wild-type behavior responses. ***A***, The five ORN activators (colored circles) and paraffin oil (dark circle) are mapped in a behavior space. *Canton S* (wild-type) larvae were tested against each odorant. Shown are the first three principal components (PCs) of a multidimensional behavior space made up of nine navigational descriptors measured at 10^−2^ concentration of odorants (RI, number of runs per trajectory, run length (toward), run length (away), run speed (toward), run speed (away), run ratio, run direction, and length/displacement). Navigational descriptors were normalized. Variances explained by PC1, PC2, and PC3 are 91.3%, 8.1%, and 0.6%, respectively. ***B***, Euclidean distances between individual combinations of odorants in the behavior space.

Since the behavioral parameter, the run ratio, correlated highly with attractive index (*r* ^2^ = 0.90), we searched for a specific example to demonstrate that odorants can affect the composition of behavior in a way that is not directly related to run ratio. At 10^−2^ concentration, 4-hexen-3-one elicits a strong attractive response from wild-type larvae (0.23 ± 0.04) compared to the weak attractive response elicited by 2,5-dimethylpyrazine (0.04 ± 0.05; Fig. 2*C*). While both odorants elicit similarly high values for run ratio (1.88 and 1.57, respectively), 4-hexen-3-one elicits significantly lower run length (144.36 ± 8.16 vs 222.75 ± 17.63) and run speed values (3.67 ± 0.09 vs 4.78 ± 0.14) compared with 2,5-dimethylpyrazine (Student’s *t* test, *p* < 0.05; Fig. 2*E–G*). To further confirm that 4-hexen-3-one and 2,5-dimethylpyrazine differentially affect the composition of larval navigation, we mapped them in a nine dimensional behavior space in which each dimension represents either the navigational index or one of eight discrete behavior elements considered in Figure 2*I*. The two odorants mapped far apart from each other (Fig. 3*A*); the Euclidean distance between 4-hexen-3-one and 2,5-dimethylpyrazine was 42.57 arbitrary units (a.u.), whereas the mean distance between all pairwise combinations of the five odorants was 31.75 ± 3.99 (mean ± SEM; Fig. 3*B*). Based on Euclidean distances between odors, the map reveals possible relationships among neurons. We note that 4-hexen-3-one maps closest to 4,5-dimethylthiazole in the behavior space (10.02 a.u.). Similarly, pentyl acetate, anisole, and 2,5-dimethylpyrazine map close together. Interestingly, Or33b (activated by 2,5-dimethylpyrazine) and Or47a (activated by pentyl acetate) are coexpressed in the same larval ORN (Fishilevich et al., 2005; Kreher et al., 2005).

### Differential navigation elicited in larvae with single functional ORNs

**Fig. 4.**
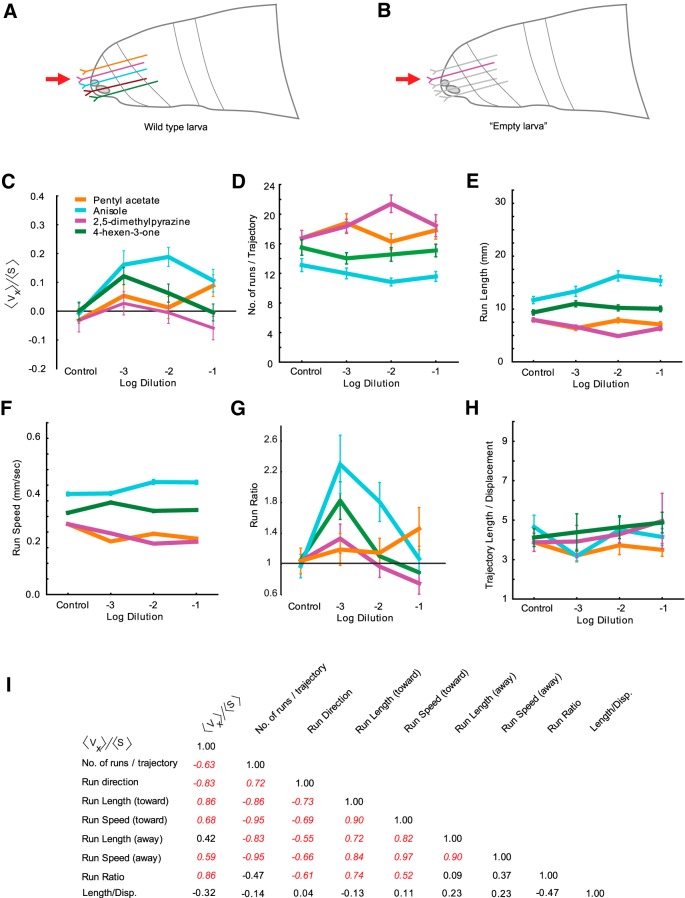
Navigational analysis of larvae expressing a single pair of functional neurons. ***A***, ***B***, Cartoons depicting a wild-type larva (***A***), in which all first-order sensory neurons are functional and an empty larva (***B***), in which only one pair of sensory neurons is functional. ***C–H***, Dose–response analysis of six navigational parameters for each odorant in the navigational assay. Odorants were tested at three different dilutions (10^−1^, 10^−2^, 10^−3^) depicted on the *x*-axis on each graph. The *y*-axes in each graph are as follows: navigational indices (*<v_x_>/<s>*) of larvae measured in response to indicated dilutions of four odorants and paraffin oil (***C***); the mean number of runs per trajectory (***D***); the mean length of runs in millimeters (***E***); the mean speed of runs in millimeters per seconds (***F***); the ratio of mean run lengths in the direction of odorant (all runs that oriented between +45^°^ and −45^°^) to mean run lengths away from odorant (all runs that oriented between +135^°^ and −135^°^; ***G***); and the mean length/displacement, defined as the total length of each trajectory divided by the total displacement of each trajectory (***H***). Each data point represents mean ± SEM (*n* = 8 assays, ∼100–120 trajectories analyzed for each condition). ***I***, Correlation matrix displaying *r* ^2^ values among various behavioral parameters tested. Values italicized and in red are statistically significant (*p* < 0.05).

Since our panel of odorants consists of strong, specific activators of single ORNs, we wished to know whether silencing all but a single pair of ORNs in a larva would affect its navigation toward the cognate odorant. A recent study (Fishilevich et al., 2005) suggested that larvae with only a single pair of functional ORNs are able to chemotax robustly toward a subset of odorants that activates it. A caveat of this study was that a simple chemotaxis index based on the distance of larva from the odor was used to compare behavior responses. Further, based on our results so far, we postulated that animals with different single pairs of functional ORNs would exhibit different compositions of navigational behavior. To address these questions, we constructed animals with single pairs of functional ORNs (Fig. 4*A*,*B*). This was achieved by exploiting the *Orco* mutation, which prevents OR trafficking to the sensory dendrite (Larsson et al., 2004; Neuhaus et al., 2005; Benton et al., 2006). *Orco* function was restored in individual ORNs by crossing animals with specific *OrX*-Gal4 drivers to UAS-*Orco* animals (Fishilevich et al., 2005). We were able to construct three different genotypes, each containing a single, functional pair of ORNs expressing either Or30a, Or42a, or Or33b/47a. For convenience, we refer to them as "*OrX-empty larva*." Since Or33b and Or47a are coexpressed in the same ORN, we chose to use the Or47a-Gal4 to construct an *Or47a-empty larva* to represent the Or33b/Or47a ORN. Due to lack of a viable Or59a-Gal4 strain, we were unable to construct an *Or59a-empty larva*.

We tested the behavioral responses of the three *empty larva* genotypes in the tracking assay. The behavior of each genotype was tested against the cognate odorant that elicits a strong physiological response from the pair of functional ORNs that it contains. *Or30a-empty larva* was tested against anisole; *Or42a-empty larva* was tested against 4-hexen-3-one. Since Or33b and Or47a coexpress in the same ORN, we tested the behavior response of *Or47a-empty larva* against 2,5-dimethylpyrazine as well as pentyl acetate separately. First, for each combination, we measured the navigational index *<v_x_>/<s>*. When tested across three different dilutions (10^−1^, 10^−2^, 10^−3^), the four odorants generated dose–response curves that varied widely from each other (Fig. 4*C*), consistent with the previous two experiments (Figs. 1*C*, 2*C* ). However, we also noted some differences. Notably, anisole generated a stronger response from *Or30-empty larva* than 4-hexen-3-one did from *Or42a-empty larva* at 10^−1^ and 10^−2^ dilutions.

Next, we extended our behavioral analysis of the three genotypes to include the five navigational parameters considered in the previous experiment. Overall, empty larva genotypes had lower run speeds compared with wild type (0.31 ± 0.005 mm/s, SEM; *n* = 1305 trajectories; vs 0.43 ± 0.012 mm/s, SEM; *n* = 1904 trajectories) and smaller run lengths compared with wild type (9.66 ± 0.53 mm, SEM; *n* = 1305 trajectories; vs 17.83 ± 1.09 mm, SEM; *n* = 1904 trajectories). This is consistent with previous observations of lower speed in *Orco* mutant larvae compared with wild-type larvae (Mathew et al., 2013). We noted that dose–response curves of *Or47a-empty larva* to pentyl acetate and 2,5-dimethylpyrazine showed similar trends, consistent with the fact that the cognate receptors for the two compounds (Or47a and Or33b) are coexpressed in the same pair of neurons. Dose–response curves for the five navigational measures revealed surprisingly large differences among responses of individual *empty larva* genotypes to their cognate odorants (Fig. 4*D–H*). Although the differences among the responses of individual genotypes were quite varied, in a correlation matrix consisting of correlation values (*r* ^2^) between individual behavioral parameters, we noticed more correlation among individual behavior parameters in this dataset when compared with correlations among parameters in the wild-type response dataset (Fig. 4*I*, Table 1), the attractive index now correlated with more (six of eight) behavioral parameters, and many more parameters correlated with each other (24 of the possible 36 combinations).

**Fig. 5.**
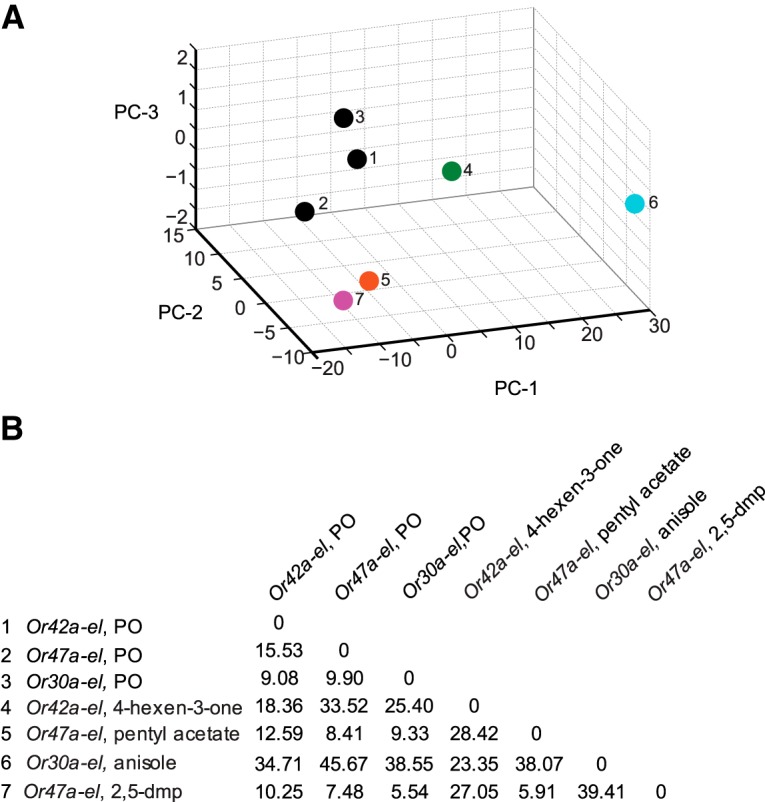
Principal component analysis of empty larval behavior responses. ***A***, The four ORN activators (colored circles) and paraffin oil (dark circles) are mapped in a behavior space. Each circle represents a different *OrX-empty larva (el) genotype*, odorant combination. Three dark circles (1–3) account for the control responses of each of the three genotypes used in this experiment. Shown are the first three principal components (PCs) of a multidimensional behavior space made up of nine navigational descriptors measured at a 10^−2^ concentration of odorants (RI, number of runs per trajectory, run length (toward), run length (away), run speed (toward), run speed (away), run ratio, run direction, and length/displacement). Navigational descriptors were normalized. The variances explained by PC1, PC2, and PC3 are 85.1%, 12.9%, and 1.5%, respectively. ***B***, Euclidean distances between individual combinations of odorants in the behavior space.

To further confirm that (1) pentyl acetate and 2,5-dimethylpyrazine elicit similar behavioral responses from *Or47a-empty larva*, and (2) odorants activating a different single, functional pair of ORNs differently affect the composition of larval navigation, we mapped the odors in a nine dimensional behavior space (Fig. 5*A*). As control, we plotted the paraffin oil diluent three times based on the responses elicited by it from each of the three genotypes used in this experiment (Fig. 5*A*, data points 1–3). We were encouraged to note that the three paraffin oil data points mapped close together (mean distance between the three combinations was 11.50 ± 2.02 (mean ± SEM)), whereas the mean distance between all pairwise combinations of the four odorants was 27.04 ± 4.96 (mean ± SEM). Pentyl acetate and 2,5-dimethyl pyrazine mapped close together in the behavior space. The Euclidean distance between the two odorants was only 5.91 a.u. (Fig. 5*B*). The remaining combination of odorants mapped far apart from each other, suggesting that the activity of individual pairs of ORNs affects larval behavior differently.

Overall, we conclude that single pairs of ORNs, when activated, instruct different navigational responses. We also note that, when we consider discrete behavioral elements, the activity of a single pair of ORNs is not sufficient to recapitulate wild-type navigational behavior.

### Wild-type larvae exhibit more variability in their behavior responses than larvae with a single pair of functional ORNs

In the course of our study, we observed that *OrX-empty larvae* were not only less responsive to their cognate odorants but also showed lower variability in their responses to odorants when compared with wild-type larvae. To confirm these observations, we compared, for wild-type larvae and *OrX-empty larvae*, both the mean changes in behavior responses as well as the variance in the responses elicited by odorants.

**Fig. 6.**
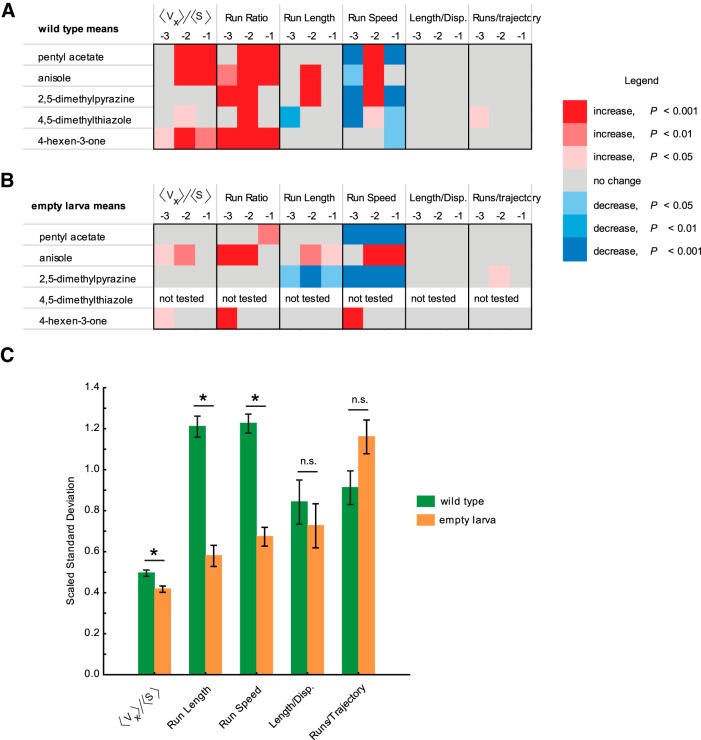
Comparisons of strength and variability of behavior responses among wild-type and empty larva genotypes. ***A***, ***B***, Heat map comparisons of statistical difference between the means of navigational parameters in response to odorants and in response to control diluent in wild-type larvae (***A***) and empty larvae (***B***). Six navigational parameters at three different dilutions (10^−1^, 10^−2^, and 10^−3^) of each odorant were analyzed. Five odorants were analyzed for wild-type larvae, and four odorants were analyzed for the three empty larvae genotypes. An arbitrary color code was assigned to visualize the statistical difference: red indicates an increase from control levels; and blue indicates a decrease from control levels. Lighter to darker shades of each color are based on an increasing level of significance. For all navigational parameters except run ratio, statistical significance was determined using one-way ANOVA followed by a Tukey’s *post hoc* HSD test. For run ratio, statistical significance was calculated with a χ^2^ test followed by a Bonferroni correction. ***C***, Mean SDs for five different behavioral parameters compared for wild-type larvae (green) and empty larvae (orange). Behavior values elicited by four odorants were used for this analysis. Each bar represents the scaled mean ± SEM. Wild-type larvae show higher variance in three of the five behavioral measures compared with empty larvae (Student’s *t* test followed by Bonferroni correction, *p* < 0.01).

To compare the mean changes in behavior responses, we considered the statistical difference between behavior values elicited by odorants and the control behavior values elicited by paraffin oil diluent. We arbitrarily assigned a color code to the statistical difference based on the increase (red) or decrease (blue) from control levels and the level of significance (light → dark shade of each color based on increasing level of significance). Mean changes in wild-type behavior response (Fig. 6*A*) and empty larva behavior response (Fig. 6*B*) were plotted for three different dilutions of test odorants (10^−1^, 10^−2^, 10^−3^). The preponderance of dark red boxes in Figure 6*A* compared with Figure 6*B* suggests that wild-type larvae show more significant behavior responses to odorants than empty larvae genotypes (for details on statistical approach, see Materials and Methods; Table 1).

To compare the variance in behavior responses among genotypes, we calculated the mean SDs for five different behavioral parameters [numbers generated across three dilutions of odorants (10^−1^, 10^−2^, 10^−3^) were averaged]. To fit the numbers generated on the same graph, SD values for each parameter were scaled (see Materials and Methods). Scaled SD values for wild-type (green) and empty larvae (orange) were plotted, and statistical significance between genotypes was determined using *t* test after applying a Bonferroni correction (Fig 6*C*, Table 1). Three of the five behavioral parameters [response index (*<v_x_>/<s>*), run length, and run speed] showed significantly higher SD values in wild-type compared with empty larva genotypes (*p* < 0.01).

Overall, we observed that wild-type larvae show stronger but more variable behavior responses to odorants compared with empty larvae that have only a single pair of functional ORNs. The stronger mean behavior response in wild-type larvae could be a result of the activation of additional ORNs at higher odorant concentrations. Higher mean variability in wild-type behavior responses could be due to spontaneous activity in nonactivated, functional ORNs or due to lateral activation of neighboring ORN channels in the larval antennal lobe, both of which are possibilities that are lacking in empty larva genotypes.

## Discussion

### Major conclusions

The major conclusion of the current study is that individual larval ORNs contribute differently to information processing in the olfactory circuit. This conclusion is based on the following experimental evidence. First, we demonstrated that strong activation of individual ORNs led to different strengths of attractive response in a simple two-choice assay (Fig. 1). We ruled out alternate possibilities that different strengths of attractive response are due either to differences in the strengths of the odor gradient or to receptor sensitivities. Next, we provided strong evidence to support the conclusion that, at the concentrations tested, the activity of individual ORNs differentially affects the composition of larval navigation (Fig. 3*A*). Finally, we show that the contributions of individual ORNs to olfactory behavior are dependent upon the presence of neighboring ORNs that are functional (Fig. 6*A–C*). In the absence of neighboring ORNs that are functional, single ORN activity elicits weaker and less variable responses to odorants. Collectively, the experimental evidence strongly supports the overall concept that individual ORNs are functionally nonequivalent.

### Conclusions in the context of available literature

This study was made possible by the recent identification of a panel of odorants that strongly activated (>150 spikes/s) single Ors in an electrophysiology analysis. In addition to strong activation, these odorants specifically activated single Ors; when tested against the entire larval receptor repertoire at 10^−4^ dilution, they elicited responses only from their respective receptors (Mathew et al., 2013). Consistent with the current study, the authors noted that individual ORN activators elicited varying behavioral responses in the larva; some odorants elicited strong physiological responses but weak behavioral responses, or weak physiological responses but strong behavioral responses. Some odorants tested in this study, 2,5-dimethylpyrazine and 4,5-dimethylthiazole, which are known to elicit strong physiological responses, seem to elicit little or no behavioral response. We suggest the possibility that some of these olfactory circuit neurons play a role in other aspects of olfactory information processing, such as inhibiting other olfactory signals or sensory integration. Another recent study (Hernandez-Nunez et al., 2015) showed that optogenetic activation of different chemosensory neurons could produce behavioral responses with distinct dynamics. Together, these studies support the conclusion that ORNs might contribute differently to the olfactory circuit and imply that there is functional individuality among a repertoire of neurons.

The collective analysis of a circuit of neurons has received due attention in the field of odor coding, because the coding of information is mainly concerned with the collective behavior of neurons (Laurent, 1996; Gaudry et al., 2012; Migliore et al., 2014). One of the main assumptions for these analyses is that neighboring neurons behave in the same way. However, our current study provides evidence for functional individuality among a class of neurons and carries implications for building more effective models of odor coding. To build a model of odor coding that can more reliably predict animal behavior, it is important to consider, in addition to the collective behavior of circuit neurons, the contributions of individual circuit neurons.

In support of the major conclusion of this study, odorants such as 2,5-dimethylpyrazine and 4,5-dimethylthiazole that elicit very weak response indices (Figs. 1*B*, 2*C*, 4*C* ) map far apart in a behavior space that considers eight other discrete behavioral elements (Fig. 3*A*). While the PCA graphs in Figures 3*A* and 5*A* effectively make the point that individual ORN activity elicits vastly different behavior responses, certain interesting observations stand out. One interesting observation has to do with the responses of larvae to 2,5-dimethylpyrazine and pentyl acetate, odorants that activate two Ors (Or33b and Or47a respectively) coexpressed in the same ORN (Fishilevich et al., 2005; Kreher et al., 2005). Wild-type larvae show very different behavior responses to the two odorants (Fig. 3*A*), while *Or47a/33b-empty larvae* respond similarly to the two odorants (Fig. 5*A*). The responses of the two Ors to their respective ligands show a difference in temporal dynamics; 2,5-dimethylpyrazine elicits a strong and long-lasting response from Or33b, while pentyl acetate elicits a strong but short-lasting response from Or47a (Montague et al., 2011; Mathew et al., 2013). Our results suggest the possibility that the activities of neighboring neurons play a role in transducing information about temporal dynamics to the olfactory circuit. In the absence of such activity, the olfactory circuit fails to distinguish between different types of temporal information supplied by the same ORN. Our results also show that the presence of functional neighboring ORNs is required for a normal response to odorants (Fig. 6*A*,*B*). One possible explanation for these observations is that the spontaneous activity of neighboring ORNs is required for appropriate transduction of information. In support of this conclusion, research in locusts has shown that baseline ORN activity is required to set antennal lobe neuron dynamics on the threshold of coherent oscillatory behavior, which in turn is important to drive behavioral responses (Laurent et al., 2001). Another possible explanation of our results is that lateral activation/inhibition of neighboring olfactory processing channels due to activity of local neurons in the larval antennal lobe is required for the appropriate transduction of information (Olsen et al., 2007; Shang et al., 2007; Olsen and Wilson, 2008). If so, inactivating lateral neuron inputs to neighboring ORNs in wild-type larvae should reduce the strength and variability of responses to odorants. It is also not clear whether similar or different neural substrates are responsible for the strength and variability of a behavior response.

Our results are consistent with a previous study that suggests that a single pair of functional ORNs is necessary and sufficient for the perception of subsets of odors (Fishilevich et al., 2005). However, inconsistent with their conclusions, our results suggest that the activity of a single pair of functional ORNs is not sufficient to elicit either similar strength (Fig. 6*A*,*B*) or variability (Fig. 6*C*) of behavior response as elicited by wild-type larvae containing all 21 functional ORNs. Differences in the setup of the behavioral assay and in analytical methods to measure the chemotaxis index along with recent advances in technology that enable the measurement of larval navigation in more detail could account for some of the inconsistencies between the two studies.

Animal behavior is notoriously variable. Variability in behavior has been described in many species, including *Drosophila* (Kain et al., 2012) and humans (Leonards and Scott-Samuel, 2005). Along with genetic sources of variation, nongenetic sources of variation in behavior responses can be used as substrates for natural selection (Hopper, 1999). The neural basis for nongenetic variability in behavior response remains unclear. In this context, we were particularly intrigued that higher variability was observed in wild-type larval population than in larval population containing a single pair of functional ORNs. Thus, the functionality of neighboring ORNs seems to influence the variability observed in behavioral responses. Further studies would be required to dissect whether spontaneous activity or lateral activation of all or some ORNs influence variability.

### Limitations of the present study

We acknowledge the limitations of certain conceptual and experimental approaches in this study. It is likely that at higher odor concentrations (≥10^−2^), odorants elicit responses from more than one ORN (Hallem et al., 2006; Kreher et al., 2008), complicating any conclusions about individual ORN contributions. However, we observe significant differences among behaviors elicited at low odorant concentrations that were shown to elicit strong, but specific responses from larval Ors (Mathew et al., 2013). Results from experiments conducted with *empty larva* genotypes that have only one functional pair of ORNs further support the main claims of this study. In these behavior assays, odor stimuli quickly form a stable odor gradient (Mathew et al., 2013). Although commonly used as a stimulus method in insect and worm olfaction studies (Rodrigues and Siddiqi, 1978; Monte et al., 1989; Zhang et al., 2005; Späthe et al., 2013; Fernández-Grandon et al., 2015), different odor gradients could elicit different levels of odor adaptation that could complicate results. We note that, unlike an adult fly, a fly larva that is normally found immersed in its natural food source has to navigate a mixture of odor gradients. Thus, the use of odor gradients in our behavior assays has ecological relevance. While it was convenient to test similar concentrations and gradient strengths of the five odorants in this study, it was more difficult to compensate for differences in the physicochemical properties of the odorants, such that, for each odorant, an equivalent number of molecules reached the larval dorsal organ (Andersson et al., 2012; Martelli et al., 2013). Thus, our results describe responses to standard dilutions of odorants and not to a defined number of odorant molecules accessible to each ORN. With recent advances in optogenetic techniques, it would be possible to precisely activate only single ORNs, and also control for the strength and duration of neuronal stimuli (Hernandez-Nunez et al., 2015).

We also acknowledge limitations in certain conclusions of our study. While it is clear that the activity of individual ORNs instruct different compositions of larval navigation, it is less clear whether individual ORNs are responsible for one or more discrete elements of navigational behavior. Our study, so far, has been unable to classify larval ORNs into distinct functional classes. Such a classification could be improved in the future by considering additional behavioral descriptors based on animal posture that were not considered in this study (Gershow et al., 2012; Luo et al., 2014). Since larval navigation is a low-dimensional behavior, we predict that the 21 larval ORNs could be classified into a small number of functional classes based on their individual contributions to navigational behavior.

Our study was restricted to first-order sensory neurons in a simple olfactory circuit of the *Drosophila* larva. Further investigation is required before our conclusions about functional individuality among a class of neurons can be broadly applied to other sensory circuits in insects and noninsect species. Recent physiological studies in mammals have revealed neuronal assemblies in which there are functional differences at the individual neuron level (for review, see Yagi, 2013). The question remains as to the origins of such individuality. Neuronal individuality could be encoded via genetic as well as nongenetic mechanisms. Differential expression of Or genes is one example of a genetic mechanism that confers individuality to ORNs. Individuality could also arise due to independent and stochastic expression of autosomal alleles, but such mechanisms are less well understood. These mechanisms together instruct differences in synaptic connection strengths that could have implications for synaptic weight distributions in theoretical models of information coding in neural circuits (Song et al., 2005)

### Final conclusions

Within its ecological niche, a larva has to navigate multiple odor gradients to reach high-quality food sources. Odorants in the environment of the larva activate one or more of its ORNs. Overall, our results suggest that individual ORN activity contribute differently to information processing in the olfactory circuit to instruct specific compositions of navigational behavior. Our analysis of functional nonequivalency among individual sensory neurons in a simple, tractable olfactory circuit has implications for the development of reliable correction factors for existing models of odor coding and for elucidating how different environmental signals are translated into different behavioral outputs.

## Synthesis

The decision was a result of the Reviewing Editor Piali Sengupta and the peer reviewers coming together and discussing their recommendations until a consensus was reached. A fact-based synthesis statement explaining their decision and outlining what is needed to prepare a revision is listed below. The following reviewers agreed to reveal their identity: Mason Klein, Elissa Hallem

The reviewers and the reviewing editor are in agreement that this paper makes an important contribution to the field by systematically analyzing the contribution of single ORNs to chemotaxis behaviors of Drosophila larvae. Although similar observations have been reported previously (see below), this paper nevertheless confirms and extends these observation in part via the use of odorants to activate ORNs. However, both reviewers felt that the writing and experimental methods/descriptions require extensive clarification, that several statements made in the paper need to be weakened to match the data, and that previous related findings need to be referenced appropriately. Since each of the reviewers noted different issues, their comments are appended below essentially unedited. Please note that the majority of the comments can be addressed by edits to the text and clarification of the writing.

Major comments:

(1) There are five odorants tested, each with a single associated ORN (at the 10^-4 level of concentration). The first time the odorants or ORNs are mentioned, please explicitly state which odor is associated with each neuron. I had to read pretty far into the paper to piece this together, and it is important to establish this at the outset. Related to this, the authors write that the concentrations gradients used for the different odors should be similar due to the volatilities, and note the average vapor pressure +/- SD; but 4.30 +/- 1.35 is quite a large range, 2.95-5.65, and presumably at least one odorant is even outside this. I feel it would be best to state directly: (i) the odorant, (ii) its Or, (iii) its vapor pressure at 25 C, (iv) its sensitivity, for all five odors, whether in a table or in the text. The primary claim of the paper depends on these values being very similar.

(2) Another point made repeatedly in the manuscript is that each odorant affects the "composition" of distinct navigational elements in a different way. This is an extremely interesting idea, but I'm not sure is supported by the evidence presented here, at least not as strongly as the authors imply. The choice of components seems a bit strange, because they are so interlocked, and it seems like only one of them ("run ratio") should have a direct effect on the navigation index v_x/s, and indeed that quantity has the highest correlation. This would be consistent with many past studies, from bacteria to C. elegans, to Drosophila larvae, where a differential run length when crawling towards or away from the stimulus leads to a biased random walk navigation. If the authors could give a specific example from their results where two odors affect the components of the behavior in some way that is not directly related to the run ratio, that would help make their claim about differential compositions.

(3) Related to the previous point, I notice the authors list run length (toward) and run length (away), and the run ratio… but for speed, only speed (toward) and speed (away) are listed, but not a speed ratio? It would be useful to include this, as it would answer a specific question about chemotaxis: do larvae modulate their speed in order to successfully navigate. If they do, it would be a convincing additional "navigation element" that is not directly tied to the run ratio.

(4) What exactly is the definition of a "Track"? It does not seem to be given in the text. Does each crawling animal have one track that lasts the entire duration of the experiment? If not all tracks are the same duration, then I find using "No. of runs / Track" as a navigation component to be problematic, because it conflates how long a run lasts and how long a track lasts. If the track duration is not constant, could this be replaced by a run duration (in seconds)? This would then give a measure of how often the animals change direction.

(5) The definition of a single run (line 134 and following) is stated in a confusing way. What does "instantaneous change" mean? The change between subsequent frames of the image acquisition? Also, based on this definition, a larva that stopped moving forward for a while, and then made a 43 degree turn - this would be considered a single continuous run?

(6) The shape of the traces in Figure 2F is pretty unique (and holds for 4 of the 5 odors, with very small error bars) - could the authors speculate on why crawling speed as a function of odor concentration would change like this?

(7) The two 3D graphs of PCA are difficult to read because the location of the points is ambiguous (a limitation of manuscripts being printed on flat paper). Please add a faint or dashed vertical line connected each of the colored dots to their PC-2/PC-1 axis point?

(8) The limitations section is good, and brings up a fair number of potential criticisms of the study, and I find it refreshing to see. However, it feels like the manuscript up to that point is written as if that section is not there. For the claims that are brought into question in the limitations section (results at higher odor concentrations, etc.), it would be best if these were not stated so strongly and so many times in the main text.

(9) The authors need to justify more how this study makes a significant conceptual advance in our understanding of odor coding over previous studies. The finding that different ORNs contribute differently to the odor code has been demonstrated previously. It was suggested by Mathew et al, 2013 (Carlson lab) based on the finding that odorants that activate different receptors elicit behavioral responses of different strengths. In particular, the strength of the ORN response did not correlate with the strength of the behavior. It was then demonstrated directly by Hernandez-Nunez et al, 2015 (Samuel lab). That paper used optogenetic stimulation of neurons to provide a clear demonstration that individual ORNs and GRNs affect larval behavior differently, without all of the issues associated with the use of actual odorants. In the present study, the authors say they are "challenging the dogma that ORNs are functionally equivalent units," but I don't think that's a fair statement given the recent papers described above. It would be more accurate to say that this paper provides additional evidence suggesting that ORNs are not functionally equivalent units. I do think it's important to confirm the findings of Mathew et al with more complex behavioral assays, and of Hernandez-Nunez et al with actual odorants. However, the present manuscript is difficult to interpret in the context of the Hernandez-Nunez paper because that paper is barely mentioned.

(11) Why are the behavioral responses weaker than those observed by Mathew et al, 2013 for the same odorants? It seems like the behavioral assay is the same.

Minor comments:

(1) The term "body bends" shows up in the significance statement and abstract, but then never again the manuscript. If this is an important navigational element it should be defined and used later, but otherwise should not be referred to. From what I can tell the behavioral analysis is based on the position of the larvae, but not their posture. Other behavioral studies of larvae (such as the Luo 2010 paper cited, also Lahiri 2011 from that same lab) look at body bending as an additional component of navigation. It doesn't have to be dealt with in the present manuscript, but it should not be mentioned (except perhaps in the limitation section, if some components based on animal posture are not being considered).

(2) Line 59, "breath" --> "breadth"

(3) A bit more detail about the preparation of the larvae would be helpful. Third instar larvae are used, but what age (approximate hours AEL)? Behavior can change significantly during that instar stage. Also, how are the larvae rinsed when taken from food (this seems important for odor experiments in particular).

(4) In Figure 2, curvature is referred to as length/displacement (and "length" is misspelled), while in Figure 4 the same quantity is called "Curve Rating."

(5) Line 26, "the proportion of discrete navigational elements" is a pretty unclear phrase. Does this mean the navigation has more of one element as less of another? The navigation elements chose for this study do not seem to be a zero sum phenomenon.

(6) Could the authors speculate in discussion about the 2,5-dimethylpyrazine and 4,5-dimethylthiazole having little effect on behavior even though they affect neuron firing? Could this perhaps be because in the circuit these neurons are involved in some kind of sensory integration, or could they inhibit other olfactory neuron signals?

(7) Empty larva genotypes had lower run speed - is this because of the Orco mutant background itself?

(8) Could speed be given in mm/s everywhere, instead of pixels/second? The latter doesn't contain as much information, since it is dependent on the camera itself, and the conversion is given in the text anyway.

(9) Line 692 (and elsewhere), captions say "5 odorants" when there are only four shown in the figure.

(10) Missing error bars in panel G (of Fig. 2 and Fig. 4). The ratio should propagate the uncertainty in its two components.

(11) In general the scale on the graphs makes it hard to see some of the traces. When the full scale isn't needed (vx/s is never above about 0.25 for example), could the scale be adjusted to better see the traces?

(12) I'm not sure what to make of the PCA analysis for odorants that don't elicit much of a response.

(13) Fig. 4: I'm confused about what the different genotypes are. I'm assuming the color-coding indicates genotype and odorant, not just odorant, but this should be clarified. I don't see genotypes mentioned at all in the figure or legend.

(14) I'm also confused about the "Or47a/Or33b-empty larva" experiment (Fig. 5A). Did the authors put both receptors back into the empty larva, or each one individually? Again the genotypes are not specified in the figures. Based on the methods, it doesn't look like "Or33b" is used at all, but this is not consistent with the main text. This needs to be clarified so that this experiment can be interpreted.
